# Culturomics Approaches Expand the Diagnostic Accuracy for Sexually Transmitted Infections

**DOI:** 10.3390/ijms221910815

**Published:** 2021-10-06

**Authors:** Ellinor Anna Wolf, Hannah Clara Rettig, Mariia Lupatsii, Britta Schlüter, Kathrin Schäfer, Dirk Friedrich, Simon Graspeuntner, Jan Rupp

**Affiliations:** 1 Department of Infectious Diseases and Microbiology, University of Lübeck, 23538 Lübeck, Germany; ellinor.wolf@student.uni-luebeck.de (E.A.W.); Hannah.Rettig@student.uni-luebeck.de (H.C.R.); Mariia.Lupatsii@uksh.de (M.L.); Kathrin.Schaefer@uksh.de (K.S.); Dirk.Friedrich@uksh.de (D.F.); Simon.Graspeuntner@uksh.de (S.G.); 2 Department of Gynecology and Obstetrics, University Medical Center Schleswig-Holstein, 23538 Lübeck, Germany; Britta.Schlueter@uksh.de; 3 German Center for Infection Research (DZIF), Partner Site Hamburg-Lübeck-Borstel-Riems, 23538 Lübeck, Germany

**Keywords:** microbiota, sexually transmitted infections, culturomics, culturing, diagnostics

## Abstract

Sexually transmitted infections (STIs) are a major health concern with clinical manifestations being acknowledged to cause severe reproductive impairment. Research in infectious diseases has been centered around the known major pathogens for decades. However, we have just begun to understand that the microbiota of the female genital tract is of particular importance for disease initiation, infection progression, and pathological outcome. Thus, we are now aware that many poorly described, partially not yet known, or cultured bacteria may pave the way for an infection and/or contribute to disease severity. While sequencing-based methods are an important step in diagnosing STIs, culture-based methods are still the gold-standard method in diagnostic routine, providing the opportunity to distinguish phenotypic traits of bacteria. However, current diagnostic culture routines suffer from several limitations reducing the content of information about vaginal microbiota. A detailed characterization of microbiota-associated factors is needed to assess the impact of single-bacterial isolates from the vaginal community on vaginal health and the containment of STIs. Here we provide current concepts to enable modern culture routines and create new ideas to improve diagnostic approaches with a conjunct usage of bioinformatics. We aim to enable scientists and physicians alike to overcome long-accepted limitations in culturing bacteria of interest to the human health. Eventually, this may improve the quality of culture-based diagnostics, facilitate a research interface, and lead to a broader understanding of the role of vaginal microbiota in reproductive health and STIs.

## 1. Introduction

Sexually transmitted infections (STIs) continue to represent a considerable global health problem with high disease incidence, causing significant morbidity and mortality [1]. Currently, more than 30 pathogens have been identified as being sexually transmitted, eight of which seem to account for the greatest burden of morbidity [2]. In 2016, the WHO estimated that there were 376.4 million new infections with one of the four most common curable STIs: chlamydial infections, trichomoniasis, gonorrhea, and syphilis. This corresponds to more than one million new infections each day [3].

While many efforts have been made to advance detection and treatment of life-threatening conditions such as HIV, curable STIs have come into focus in recent years, as they are associated with a vast array of not only acute symptoms but also long-term health consequences. These include various genital or urethral symptoms, ailments of the upper genital tract, reproductive and pregnancy complications, and extra-genital manifestations. There is also evidence that curable STIs can increase both HIV susceptibility and transmission rates [4]. However, a large proportion of STIs present asymptomatically, thus remaining undetected and going without timely treatment. Table 1 lists usual bacterial etiologies of genital tract infections [5]:

*C. trachomatis* infections are the largest fraction of bacterial STIs and are asymptomatic in approximately 70% of women and 50% of men [6]. Acute genital chlamydial infection (Serovars D-K) can cause conjunctivitis and urethritis in males and females, cervicitis in females, and proctitis in males. Without treatment, the pathogen can ascend from the lower to the upper genital tract. Among the possible complications in females are adnexitis, endometritis, and pelvic inflammatory disease (PID), which can entail chronic pelvic tenderness or pain, tissue scarring, tubal factor infertility, ectopic pregnancies, and, in rare cases, perihepatitis (Fitz-Hugh-Curtis syndrome). In males, the referred infection can result in epididymitis and orchitis and therefore possible infertility. In both sexes, *C. trachomatis* infection is associated with Chlamydia-induced reactive arthritis and may also be involved in the pathogenesis of chronic undifferentiated spondylarthritis [7].

## 2. Importance of Understanding Microbiota in STIs

The approach to STI research has long been shaped by the public health notion of combating spread of infection on the population level. Indeed, fundamental variables such as socioeconomic factors, sexual behavior, knowledge, and health care structure and utilization are important determinants in understanding STI epidemiology [8]. Research on transmission dynamics as well as pathogen infection mechanisms and their interaction with the host have been pivotal in developing treatment and preventive strategies and shaping public health policies.

During recent years, the role of site-specific microbial communities in individual health and susceptibility to disease has gained a new level of recognition. Knowledge about the microbial composition in a given body location had previously been limited or skewed when using classic culture and identification methods, since a large fraction of the total bacterial diversity are not culturable under standard laboratory conditions [9] and are therefore potentially not yet identified. 

Like other areas and niches of the human body, the vagina has been shown to harbor an intricate community of bacteria [10] that is key to preventing infections with pathogenic organisms [11]. Conversely, disruption of this mutualistic relationship, as seen with bacterial vaginosis (BV)—a condition characterized by a vaginal environment low in *Lactobacillus* spp. with a high abundance of anaerobic species and an elevated pH value—has been associated with an increased disposition to acquiring a variety of STIs [12,13,14]. Additionally, it has been linked to vulvovaginal candidiasis, urinary tract infections, the development of PID, and complications during pregnancy [15,16,17,18,19].

Ravel et al. (2011) was the first study to cluster the vaginal microbiota into five major categories according to their bacterial composition, as determined using 16S rRNA sequencing [10]. Group I was defined as being dominated by *Lactobacillus crispatus*. The other group-dominant species were *L. gasseri* (Group II), *L. iners* (Group III), and *L. jensenii* (Group V). The most diverse communities were subsumed under Group IV and exhibited greater proportions of anaerobic taxa such as *Prevotella*, *Dialister*, *Atopobium*, *Gardnerella*, *Megasphaera*, *Peptoniphilus*, *Sneathia*, *Eggerthella*, *Aerococcus*, *Finegoldia*, and *Mobiluncus*. While Group I had the lowest median pH (4.0 ± 0.3), Group IV was found to have the highest median pH (5.3 ± 0.6) [10].

These groups, later termed “community state types” (CSTs), were found to be associated with ethnicity and subject to temporal dynamics, meaning that community composition varied in individuals over time, with constancy or fluctuation depending on factors such as the CST itself, menstrual cycle, and sexual activity [20]. Other studies have provided evidence of the existence of further CSTs, such as one type with a high abundance of *G. vaginalis* [21,22,23,24,25].

## 3. Role of Microbiota-Driven Microenvironmental Conditions in STIs

Many of the qualities that characterize a healthy vaginal microbial community and are believed to protect against STIs have been attributed to the presence of lactobacilli. One such attribute is the production of lactic acid, lowering the vaginal pH value to an estimated level of 3.5 ± 0.3 [26]. It has been shown, although mainly in vitro, that a variety of different pathogens in the reproductive tract, including *C. trachomatis*, are inactivated by either lactic acid itself or acetic conditions in general [27,28]. When the ability of lactobacilli culture supernatant to inactivate infectivity of *C. trachomatis* elementary bodies (EBs) in HeLa cell culture was compared among *L. crispatus*, *L. gasseri*, and *L. vaginalis*, *L. crispatus* was shown to have the strongest anti-chlamydial effect [29]. While lactic acid alone did have a strong anti-chlamydial effect when buffered at pH 4 [29], there are indications that other factors besides pH value alone are involved. Witkin et al. reported that vaginal samples dominated by *L. crispatus* were higher in D-lactic acid than samples dominated by *L. gasseri* or *Gardnerella* spp. [30]. A high concentration of L-lactic acid and an elevated ratio of L-lactic acid to D-lactic acid (distinctly elevated in communities dominated by *L. iners*, *L. jensenii*, *Gardnerella*, or *Streptococcus*) was correlated with increased levels of the extracellular metalloproteinase inducer (EMMPRIN, CD147). This is a protein located on human host cells and in the extracellular matrix, serving as an inducer of matrix metalloproteinase 8 (MMP-8) [30]. MMP-8, among other entities, and seeming to play a role in inflammatory processes and pathogen invasion of the upper genital tract during pregnancy [31].

It has also been suggested that *L. crispatus* possesses direct immunomodulatory properties. In cell culture, *L. crispatus* has been shown to inhibit infection with *Candida albicans* by interfering with levels of human β-defensins as well as Toll-like receptors 2 and 4 and cytokine IL-8 expression [32]. When three-dimensional vaginal epithelial cell aggregates were colonized with *L. crispatus* and *L. iners* to measure induction of specific signatures in host immune response, *L. crispatus* did not induce significant pro-inflammatory cytokine secretion, while *L. iners* was shown to elicit significant epithelial cell activation by inducing pattern-recognition receptor (PRR) signaling [33]. In vivo, such activation is linked to alteration of mucosal immune barrier properties, as seen with BV-associated bacterium *Atopobium vaginae*, and might influence the susceptibility to and progression of STIs [33]. With regards to chlamydial infectivity and course of disease, Rizzo et al. (2015) was able to demonstrate that *L. crispatus* and its supernatant caused a reduction in the pro-inflammatory cytokines IL-6, IL-8, and TNF-α while increasing expression of the anti-inflammatory cytokine IL-10 in *C. trachomatis*-infected cell cultures [34].

Other factors under suspicion of influencing infection in the urogenital tract are metabolites produced by members of the microbiome [35]. Apparently, a dysbiosis with presence of microbial indole producers such as *Prevotella intermedia* and *P. nigrescens* [36] is able to rescue *C. trachomatis* from host-induced tryptophan depletion [37].

In a study examining the association of BV with the presence of specific bacterial species, women with high levels of *L. iners* could be either BV-positive or BV-negative, and the presence of *L. iners* was not associated with the absence of BV, in contrast to the presence of *L. crispatus* [38]. Since *L. iners* has a considerably smaller genome size than *L. crispatus* and reduced metabolic capacities—for instance, it completely lacks the ability to produce D-lactic acid—it relies more on exogenous nutrient sources. It might therefore be more sensitive to environmental changes, rendering *L. iners*-dominated vaginal communities likely less stable than those dominated by *L. crispatus* [39]. This would be in concordance with the suggestion that *L. iners* could promote transition between healthy vaginal states and BV [28].

Further important mechanisms affecting how lactobacilli can impede pathogen colonization of the vaginal tract are co-aggregation and competition during pathogen adhesion to host cells. Mastromarino et al. (2014) explored the ability of *L. brevis* and *L. salivarius* to interfere with *C. trachomatis* in different phases of its developmental cycle in cell culture [40]. When co-cultured with HeLa cells, both *Lactobacillus* species were able to co-aggregate with *C. trachomatis* elementary bodies (EBs) and competed with *C. trachomatis* during adsorption, thereby significantly reducing chlamydial recovery compared to a co-culture of HeLa cells with *C. trachomatis* alone. Even when lactobacilli were added to the culture after chlamydial entry into the host cells, a significantly reduced number of chlamydial IFU could be observed compared to control [40]. A similarly constructed in vitro study has likewise shown that the ability of *N. gonorrhoeae* to adhere to and invade human endometrial epithelial cells was significantly reduced by the presence of *L. jensenii* and *L. gasseri* [41].

## 4. Consequences for STI Culture Diagnostics

The advent of genetic sequencing techniques has brought an indispensable tool to STI diagnostics. However, classic culture techniques continue to play a pivotal role in clinical routine. In addition to being more readily available and more affordable—factors not to be underestimated—culture techniques have the critical advantage of revealing more phenotypical traits, which are of interest to clinical decision-making. However, due to numerous limitations inherent in the cultivation process, a large amount of information on different microbial communities in the genital tract, such as their interdependencies, is bound to remain elusive.

While current methods routinely employed to diagnose STIs may be inadequate to identify the large spectrum of pathogenic factors involved, recent large-scale culturomics approaches have been introduced and shown to be of great benefit for the understanding of microbiota in disease. Those culturomics methods have, however, not yet been adapted to the field of STIs. In the subsequent sections, we provide an overview of current culture methods and modalities in vaginal diagnostics, as well as their limitations and ambiguities. We also present novel concepts and seek strategies to improve cultivation-based diagnostics, both by optimizing the manual sample processing and though usage of bioinformatic tools.

## 5. State-of-the-Art Culture Diagnostics

### 5.1. Sample Collection and Transportation

Samples for cultivation of microbes from the female urogenital tract are routinely taken using a swab. Swabs from the cervix should be collected without touching the vaginal mucosa (leading to the necessity of using specula), as should swabs from the vagina without touching the vaginal introitus to minimize contamination by the resident microbiota, especially when clinically relevant results involving anaerobes are required. For the transportation of anaerobic specimens, anaerobic transport media in containers that exclude air are available. The best system is considered to be oxygen-free transport tubes or vials with pre-reduced and anaerobically sterilized (PRAS) medium, so called ATMs (anaerobic transport media). Special requirements for specific pathogens, such as *C. trachomatis* or *N. gonorrhoeae*, should be considered [42].

**Figure 1:**
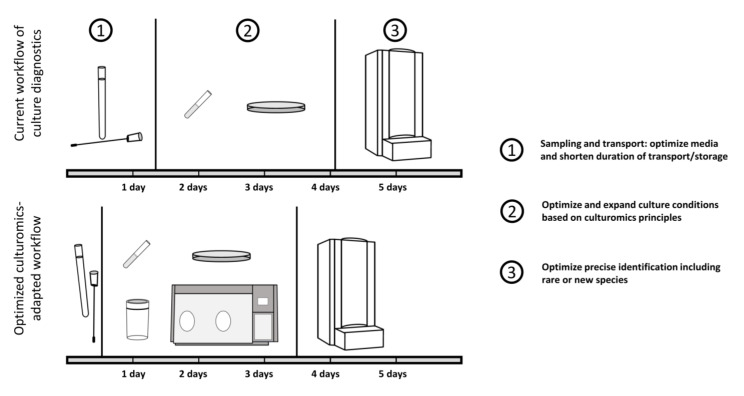
Schematic workflow of routine diagnostics with suggestions for optimization. Current routine diagnostics are suffering from a prolonged duration from sampling to cultivation, with culture conditions enabling only a limited number of microbes to be identified.

All samples from the vaginal tract regardless of the suspected pathogen should be processed as soon as possible (ideally within two hours at the latest), while storage up to 24 h is accepted (Figure 1). During transportation and storage, specimen samples, especially if they are suspected to contain clinically relevant anaerobes, are kept at room temperature [42]. Fastidious bacteria such as *N. gonorrhoeae* require immediate processing, because even delays beyond 6 h result in significant loss of viable organisms, though less so if the samples are refrigerated [43,44]. This makes optimal transportation conditions very important.

### 5.2. Sample Processing

Swabs that will be inoculated onto only one or two plates can be rolled directly across the agar surface, starting with the least-inhibitory medium. If a Gram stain is requested (mandatory for anaerobic diagnostics, e.g., diagnosis of BV) or if numerous media must be inoculated, the swab can be vortexed in sterile saline, trypticase soy, or thioglycolate broth for homogenization, and drops of the suspension can be used to prepare the slide and inoculate the plates [42]. If swabs are provided in liquid transport medium, they can be vortexed directly in the medium.

Chocolate agar plates are always incubated in 5% CO_2_, blood agar plates may be incubated in either air or CO_2_ (depending on the requirements of the organism selected for recovery), and selective agar plates are best incubated in air. If a pathogen with specific requirements is suspected, an appropriate medium should be chosen. Ideal temperatures for bacterial growth are 35 to 37 °C [5].

Specimens for anaerobic culturing should be processed as soon as possible after arrival in the laboratory, and media should be immediately incubated in an anaerobic environment (within 15 to 20 min) after inoculation [42]. Incubation in anaerobic containers is acceptable [42,45]. There are several different systems of anaerobic containers available, which are based on either an in-jar chemical consumption of oxygen and release of CO_2_ within hours or the exchange of gas by rapidly evacuating air and replacing it with an anaerobic atmosphere within minutes. Most anaerobes grow more slowly than aerobic or facultative bacteria. Therefore, jars or boxes should not be opened in air before 48 h of incubation to prevent killing of organisms in the logarithmic growth phase. Anaerobic cultures should be incubated for at least five days before being reported as negative. For some fastidious anaerobes, an even longer incubation (up to seven days) is necessary [42].

### 5.3. Examination and Identification of Bacteria

Once colonies have grown on the agar plates, potential pathogens requiring identification and antimicrobial susceptibility testing must be differentiated from contaminants [42]. Aids to interpretation are the specimen source, the relative quantities of each isolate, correlating culture results with Gram-stained smear results, and recognizing usual pathogens and contaminants from the respective specimen sites [5]. Initial examination should include magnifying aids to differentiate apparently similar colony morphologies and to discern tiny colonies, especially when examining colonies grown in anaerobic conditions. Some organisms can be identified quickly and cost-effectively based on colony (e.g., pigmentation or fluorescence under UV light) and Gram-stain morphology, motility, and biochemical spot tests, while others require more extensive methods. Testing susceptibility of bacterial isolates to different antibiotics is used to help with further identification but takes 24 to 48 h and is not very reliable in the case of anaerobic bacteria, some of which can have quite unpredictable susceptibility patterns [42,46].

For laboratories without molecular assessment capabilities, the VITEK^®^ 2 system is a good choice for identification and antibiotic susceptibility testing of bacterial isolates, and its ANC ID card has acceptable performance for the most common clinically relevant anaerobic bacteria [47]. If molecular assessment is possible, matrix-assisted laser desorption/ionization time of flight mass spectrometry (MALDI-TOF MS), or sequencing of genetic markers such as fragments of the 16S rRNA gene are recommended [48] (see also Box 1).

Box 1 **Function of modern tools for microbial species identification.**

The **Vitek 2 XL** (bioMérieux, France) is an automated system for identification (ID) and/or antibiotic susceptibility testing (AST) of bacteria and fungi. Therefore, each microorganism must be isolated and grown in pure culture on agar. Cards for ID or AST of the microorganism are inoculated with suspension and loaded into the VITEK 2 XL device. ID cards contain biochemical substrates for identification of the microorganism, while AST cards provide dilution series of several antibiotics for determination of the minimum inhibitory concentration. The Vitek 2 XL system has an optical unit, which reads out the substrate reactions (ID card) or growth of the microorganism (AST card) after 4-13 h of incubation. The obtained reaction patterns are compared to a database, which finally provides the identification or an antibiogram of the tested microorganism [49].

The **MALDI Biotyper MBT smart system** (Bruker, MA, USA) is used to identify bacteria, yeast, or mold by a matrix-assisted laser desorption/ionization time of flight (MALDI-TOF) mass spectrometry. The microorganism of interest is determined by its specific protein pattern. Therefore, a single colony of a pure culture is transferred onto a target and covered with HCCA (α-Cyano-4-hydroxycinnamic acid) matrix. Inside the system, the target is hit by a pulsed ultraviolet laser in vacuum conditions. The matrix absorbs the energy of the laser, which is converted into thermal energy, and transfers the sample into the gas phase. The molecules of the sample are ionized, and their masses are analysed by separating them by their mass to charge ratio, which is determined by the time it takes for the ions to reach the detector (TOF spectrometry). Final identification of the microorganism is performed by comparing the obtained spectra to those of the manufacturer’s database [50,51].

In contrast, **sequencing** strategies can be employed to identify species from a pure culture as well as directly from a sample, circumventing the cultivation step. In the case of a pure culture, a fragment of the bacterial 16S rRNA gene is amplified and sequenced by sanger sequencing. The resulting sequence is then compared to a reference database for classification. For direct taxa identification from samples, next-generation sequencing methods are applied, which enable high-throughput analysis of DNA content of a large number of samples in parallel. In the case of parallel 16S rRNA gene sequencing, partial 16S fragments are amplified from the pool of bacteria residing within a sample and are taken as proxy for the presence of the respective bacteria. In addition, whole-genome shotgun sequencing can be employed directly from a sample, which not only provides the bacterial composition of a samples but enables a full coverage of bacterial genomes and thus a broader scope of information about the microbiome [52,53].

## 6. Limitations and Developments in Routine Culture Diagnostics

Correct sampling is the first line of successful cultivation. Although many known clinically relevant bacteria are easy to maintain during transport, further suitability of transportation systems beyond expected pathogens has often been neglected in clinical settings and studies. Thus, many studies [43,44,54,55,56,57,58,59,60,61] have been performed to establish which swab-medium-tube system is the most suited for maintaining the viability and stability of various fastidious and non-fastidious bacteria under different transport and storage conditions.

As transport devices for obligately anaerobic bacteria, vials or tubes with anaerobic atmosphere and special oxygen-absorbing media to maintain organism viability should be used. Here, glass vials are superior because plastic tubes are known for oxygen seepage [54,59]. Anaerobic transport media (ATMs) contain buffered mineral salts for pH moderation, sodium thioglycolate, and cysteine to provide a reduced environment as well as resazurin as an indicator of oxygen exposure to the medium. ATMs provide an environment which maintains the viability of most microorganisms, including obligate anaerobes, without significant multiplication (especially not of coliforms) and allows for dilution of inhibitors present in clinical material. Media in liquid form are preferentially used over gel forms, due to providing a better recovery of fastidious bacteria and usability in automated inoculation systems [60].

Thus, cultivation of microbes already starts with selection of the transport medium and its ability to maintain viability of fastidious bacteria and prevent overgrowth of non-fastidious species. However, further important factors complicate cultivation efforts in clinical settings. Timely sample transportation is most difficult due to the organizational paradigm of most clinical transport structures, which are mainly based on efficacy more than urgency, and due to centralization of specialized labs, which leads to elongated transfer times. If samples are refrigerated, storage even up to 48 h is acceptable [42,59]. It has, however, been shown that consideration of storing temperature is very important, with 4 °C or even refrigeration being the prerequisite for such a long viability of the tested fastidious microbes [56,58,60,61]. Independently, some bacteria, such as *Atopobium vaginae*, show a decrease in viability even under such optimized circumstances [55], and it remains to be seen how far these results can be transferred to yet uncultured bacteria, which may become relevant in the future. Additionally, presently, unknown microorganism interactions within the mixtures used could impact viability in ways that are not yet understood [55]. Together with the risk of overgrowth by fast-growing facultative bacteria [55,57], these findings are very important for mixed infections or pathogens on sites with commensal bacteria, because poor sample handling could distort recultivation of microbes of interest.

These thoughts leave us with the question if an all-round transport system for vaginal swab samples is even possible. It is most certainly preferable, because the bacterial composition of the vaginal tract should be sampled and transported as exactly as possible to ensure detection of rare infectious particles, whose viability may depend on members of the microbiota. All studies mentioned above compare different transport systems in their entirety. This makes it difficult to establish standards for single components of transport systems and evaluate which swab, which medium, and which vessel is the best suited for vaginal samples. Therefore, the best way of avoiding bias by transport systems is direct inoculation of the specimen, which is not practicable in most cases. Consequently, transport and storage optimization will foster broad spectrum cultivation efforts.

There is always a selection based on media and incubation conditions even if non-selective culture media are used. Different nutrients, temperature, pH value, and oxygen levels affect different bacteria in different ways, enabling better growth of some and reduced growth of others. It is currently recommended to incubate vaginal and cervical swabs under aerobic conditions on standard media, that is, blood agar and various selective agars depending on the suspected pathogen. As a result, the number of potential microbes identified is limited to those with the respective growth conditions. 

However, as the vaginal microbiome partially consists of anaerobes, most of those would be neglected by incubating under aerobic conditions only. There are several different anaerobic culture systems available. Doan et al. (1999) compared the ability of three anaerobic culture systems: an anerobic chamber and two different systems with chemically generated anaerobic atmospheres (GasPak and AnaeroPack). The anaerobic chamber and the GasPak system showed the highest proportional recoveries, but each system excelled in recovering different bacterial genera. Additionally, the recoveries by the tested anaerobic culture systems varied considerably from sample to [45].

Anaerobic bacteria are especially difficult to examine. Because of their sometimes-slow-growing nature, they appear as tiny colonies on the agar plates and are therefore easily overlooked. This might be one of the reasons, along with improper sample handling and inadequate incubation conditions, why pathogenic anaerobes have been previously neglected and are not defined as common or important pathogens. However, modern MALDI-TOF MS (Box 1) systems precisely identify anaerobic bacteria [62]. With a very small amount of biomass needed for correct identification, this provides an early identification for anaerobes, very often directly from the primary culture plates, without additional subculturing [42]. Notably, growth conditions, sample preparation, and pretreatment are critical to consider for optimal results [63].

Hereafter (Table 2), we present an overview of unspecific methods for bacterial identification and compare their advantages and disadvantages [42,46,47,48,62,63,64,65,66,67,68,69,70]:

## 7. Culturomics as a New Tool to Improve Diagnostic Standards and Treatment Options

Recent studies using molecular methods have shown that many organisms implicated in vaginal health and disease have not been recovered in culture yet [71,72]. New anaerobic species continue to be identified at an increasing rate due to new amplicon-based and metagenomic microbiome analysis, showing that anaerobes are involved in more types of infectious processes than were previously suspected based on culture methods alone. Browne et al. (2016) showed that culturomics approaches can lead to the discovery and isolation of such not-yet-recovered novel species from human samples [73]. Culturomics is an approach in which extensive assessment of the microbial composition is made possible by high-throughput culturing [74]. However, currently recommended agar media, especially selective ones, focus on the detection of known pathogens of the vaginal tract and neglect thus-far-unknown or uncultured bacterial species, with a possible impact on health and disease. While laboratory processing must adapt to include more molecular methods, these bacteria can only be appropriately addressed in combination with culture-based work.

Anaerobic microbiology is not routinely performed in many laboratories because of technical and financial reasons. However, due to better isolation and identification possibilities, the spectrum of anaerobic bacteria isolated is increasing [42]. Consisting of multiple culture conditions combined with the rapid identification of bacteria, the culturomics approach has enabled the culturing of hundreds of new microorganisms that are associated with humankind, providing exciting new perspectives on host–bacteria relationships [68]. Diop et al. (2019) proved that this approach is also possible for vaginal samples by describing a new strictly anaerobic species named *Collinsella vaginalis* from a patient suffering from BV [75]. As more information becomes available, the use of newer molecular tools may be necessary to complete anaerobic microbiology. Additionally, the use of total laboratory automation has shown a significant increase in cultivation of some non-typical and rarely cultured bacterial species from urine samples, which suggests that previously neglected species may be relevant pathogens [76]. Although it is the most expensive method, use of an anaerobic chamber allows for all sample manipulations and incubation without interrupting the anaerobic atmosphere and is, therefore, the best method to ensure viability of fastidious, slow-growing, and strictly anaerobic bacteria [42].

As the vaginal tract is predominantly microaerophilic [77], it might be interesting to investigate neither aerobic (air or CO_2_) nor anaerobic (containers or chamber) incubation but rather microaerophilic and hypoxic environments or break the aerobic/anaerobic bacterial culture dichotomy completely. On that note, Dione et al. (2016) introduced the quasi-universal R-medium on the basis of Schaedler agar supplemented with the antioxidants ascorbic acid, glutathione, and uric acid. Of the tested 276 different bacterial species (some of which are common in the vaginal tract), 82 strictly and 148 facultative anaerobic species, 31 aerobic species, 7 microaerophilic species, and all tested yeast species grew in air on the R-medium [78]. Hemin and α-ketoglutarate were added to increase culture of fastidious species, which led to the additional cultivation of *Eikenella corrodens*, *Haemophilus influenzae*, *Haemophilus parainfluenzae*, and *Legionella pneumophila*. Only two of the tested fastidious species (*Mycobacterium bovis* and *Mycobacterium tuberculosis*) could not be cultured on R-medium [78].

All those efforts are aiming to increase cultural recovery of fastidious microbes. To culture previously uncultured species, the work of Weimann et al. (2016) might hasten the development of new media and predict optimal incubation conditions. The authors introduced Traitar, a software that can phenotype microbial community members based on single amplified genomes, genomes from metagenomes, and genomes from microbial isolates [79]. More elaborate analysis of metagenome-based reconstruction of yet uncultured bacteria with implication on vaginal health has been suggested to further aid development of suitable culture conditions. An example of this strategy is provided by a recent study describing the new candidate species metagenome Candidatus *Lachnocurva vaginae* [80]. Thus, beyond general improvements and wet lab methodical variability, combination with genetic approaches are making culturomics efforts successful in increasing clinically relevant knowledge of the role of microbiota in sexually transmitted diseases. It is important to state that introducing such efforts to diagnostic laboratories will not be feasible without solving another current challenge, which is laboratory automation [81], as a prerequisite to reduce hands and time and increase parallelization of procedures. Thus, by combining automation and introduction of culturomics approaches to laboratories, reduced running costs are coming along with increased value of information for physicians.

Culturomics approaches can, however, not only aid in culture-based diagnostical standards by enabling cultivation of novel pathogenic microbes, because culturomics is also a tool to better understand the important contribution of commensals to vaginal health. Consequently, both patient-specific treatment strategies and probiotics are more and more used to prevent and treat complicated (e.g., recurring) infectious diseases. While probiotics are available for treatment of BV, there is a lack of knowledge about the person-specific interdependencies within the microbiota and between the microbiota and the host. However, such knowledge is a very important factor, considering security and quality management of probiotic treatment. Knowing about the actual commensal colonization of a patient suffering from vaginal disease is of utmost importance for appropriate patient-specific treatment. In combination with sequencing-based assessment of the vaginal microbiota, culturomics approaches may facilitate patient-specific probiotic strains useful for recolonization of the vagina and, thus, reestablishment of the original health state without the need of artificial microbial species. Furthermore, culturomics provide a platform for physicians and researchers to address vacancies in current molecular understanding of diseases, foster a better understanding of how microbiota interact with each other, the host, and pathogens and which metabolic processes are in the end required to recover health and fight disease.

## Figures and Tables

**Table 1:** Genital tract morbidities and their bacterial etiologies.

Genital Tract Infection	Usual Bacterial Etiologies
Ulcers	*Haemophilus ducreyi*, *Chlamydia trachomatis* (Lymphogranuloma venereum), *Treponema pallidum*, *Klebsiella granulomatis*
Vulvovaginitis, cervicitis	*Neisseria gonorrhoeae*, *C. trachomatis*
Bacterial vaginosis (BV)	Overgrowth of vaginal microbiota with anaerobic and facultative endogenous bacteria
Endometritis	Enterobacteriaceae, streptococci (groups A and B), enterococci, mixed anaerobic microbes
Salpingitis, oophoritis	*N. gonorrhoeae*, *C. trachomatis*, mixed aerobic and anaerobic microbiota
Pelvic abscess following infection	Mixed aerobic and anaerobic microbes

**Table 2:** Strengths and weaknesses of different methods for bacterial identification.

Identification Method	Strengths	Weaknesses
Morphology (colony and Gram-stain)	very low cost and time investment	identification only possible for some species examiner-dependent
Biochemical spot tests	very low cost and time investment	low reliability for anaerobes
Antibiotic susceptibility testing	important information for clinical decision making	takes 24 to 48 h low reliability for anaerobes
Automated systems such as VITEK^®^ 2 (see also Box 1)	automated identification plus antimicrobial testing	high costs
MALDI-TOF MS (see also Box 1)	early and reliable identification	initially high cost of instrument sample preparation influences spectrum quality
Partial 16S rRNA gene sequencing analysis (see also Box 1)	reliable identification	time and costs
Metagenomics/next-generation sequencing (see also Box 1)	no bacterial growth necessary discovery of new, uncultured taxa association of microbial signatures with diseases	time and costs limited discrimination between live bacteria and transient DNA results depend on used methodologies

## Data Availability

Not applicable.
